# Metabolic Syndrome Increases the Risk for Knee Osteoarthritis: A Meta-Analysis

**DOI:** 10.1155/2016/7242478

**Published:** 2016-10-11

**Authors:** Huajun Wang, Yanmei Cheng, Decheng Shao, Junyuan Chen, Yuan Sang, Tao Gui, Simin Luo, Jieruo Li, Chao Chen, Yongguang Ye, Yong Yang, Yikai Li, Zhengang Zha

**Affiliations:** ^1^The First Clinical College, Jinan University and Department of Orthopedics, The First Affiliated Hospital, Jinan University, Guangzhou 510630, China; ^2^The First Affiliated Hospital of Sun Yat-sen University, Guangzhou 510080, China; ^3^Department of Orthopedics, The Third Hospital of Hebei Medical University, Shijiazhuang 050051, China; ^4^Department of Orthopedics, School of Traditional Chinese Medicine, Southern Medical University, Guangzhou 510515, China; ^5^Department of Orthopedics, Guangzhou Orthopedic Hospital, Guangzhou 510045, China; ^6^Department of Orthopedics, Xingtai People's Hospital, Xingtai, Hebei 054031, China

## Abstract

*Background*. Studies revealed that metabolic factors might contribute substantially to osteoarthritis (OA) pathogenesis. There has been an increasing interest to understand the relationship between knee OA and the metabolic syndrome (MetS). The purpose of this study was to explore the association between metabolic syndrome and knee osteoarthritis using meta-analysis.* Methods*. Databases, including PUBMED, EMBASE, and the Cochrane Library, were searched to get relevant studies. Data were extracted separately by two authors and pooled odds ratio (OR) with 95% confidence interval (CI) was calculated.* Results*. The meta-analysis was finished with 8 studies with a total of 3202 cases and 20968 controls finally retrieved from the database search. The crude pooled OR is 2.24 (95% CI = 1.38–3.64). Although there was significant heterogeneity among these studies, which was largely accounted for by a single study, the increase in risk was still significant after exclusion of that study. The pooled adjusted OR remained significant with pooled adjusted OR 1.05 (95% CI = 1.03–1.07, *p* < 0.00001). No publication bias was found in the present meta-analysis.* Conclusions*. The synthesis of available evidence supports that metabolic syndrome increases the risk for knee osteoarthritis, even after adjustment for many risk factors.

## 1. Introduction

Knee osteoarthritis (KOA) is a prevalent chronic joint disease affecting about 28% of the population in the USA at 45 years of age. It is estimated to rise up to 37% in adults aged over 65 years [1] within the next years. KOA affects joint tissues, including synovium, ligaments, tendons, muscle, and subchondral bone, causing cartilage and osteophyte formation at joints, eventually leading to arthralgia, stiffness, and limitations in the articular function, severely affecting patients' physical functioning and quality of life [2, 3]. KOA is a heterogeneous disease, and risk factors include sex, age, mechanical factors, or obesity. Understanding the role each of the risk factors plays is important for KOA prevention.

Recently, studies revealed that metabolic factors might contribute substantially to OA pathogenesis [4]. There has been an increasing interest to understand the relationship between knee OA and the metabolic syndrome (MetS). Some studies supported the link between OA and characteristics of MetS, including hypertension, type 2 diabetes, and dyslipidemia [5, 6]. Additionally, several studies were inclined to recognize metabolic phenotype to be a common subtype of OA. Some studies support MetS as a contributing factor to an increased risk of OA. However, literature results are controversial as there are other groups that do not support this belief. The purpose of this study was to provide a summary and analysis of published studies based on the association between OA and MetS.

## 2. Materials and Methods

### 2.1. Search Strategy

The authors did an intensive search on PUBMED, EMBASE, and the Cochrane Library for published studies until June 3, 2015. Relevant available articles were obtained using key words such as ("Osteoarthritis"[Majr]) AND "Metabolic Syndrome X"[Majr], “osteoarthritis metabolism syndrome”, and “osteoarthritis metabolic syndrome”. No language restrictions were applied. A reference list of relevant papers was also screened.

### 2.2. Study Selection Criteria

A study was included if it met the following inclusion criteria: (1) It is cohort, case-control, or cross-sectional study investigating the association between MetS and risk of OA; (2) relative risk (RR) or odds ratios (ORs) and the corresponding 95% confidence intervals (CIs) could be directly extracted or calculated from the available data; and (3) if there is duplicated data, the larger sample size was adopted. Two investigators independently applied the selection criteria to each reference identified by the search strategy. Any discrepancies regarding eligibility or quality were resolved by the third reviewer.

The following information was collected from each available article: year of publication, ethnicity of the studied population, numbers of cases and controls, mean age of case and control groups, and MetS criteria and OA criteria adopted adjusted OR and adjusted variables. All articles were independently reviewed by two investigators. Data was extracted independently and entered into separate databases. Results were compared and any discrepancy was resolved by the third investigator as well.

### 2.3. Definition of Metabolic Syndrome

The National Cholesterol Education Program-ATPIII (NCEP-ATPIII) definition was used to define metabolic syndrome (MetS) [14]. MetS is defined as the presence of any three out of five components: that is, abdominal obesity ≥102/88 cm (western, male/female) and ≥90/85 cm (eastern, male/female) [8, 15]; hypertriglyceridemia ≥150 mg/dL; low high density lipoprotein cholesterol (HDL-C) <40 mg/dL in men and <50 mg/dL in women; high blood pressure ≥130/85 mmHg or use of antihypertensive medication; or high fasting glucose ≥100 mg/dL or being under treatment for diabetes.

### 2.4. Definition of Knee Osteoarthritis

Kellgren and Lawrence grade (K-L grade) classified KOA into four grades (0, normal, to 4, severe). Radiographic knee OA is defined as K-L grade 2 or above [16]. The American Rheumatism Association (ACR) proposed an alternative definition for OA based on a clinical or self-reported approach [17].

### 2.5. Statistical Analysis

The crude pooled OR was calculated. Adjusted ORs controlling for potential confounding factors in the greatest degree were also extracted whenever available. The adjusted OR was converted by using the natural logarithm and the SEs and their corresponding 95% CIs were calculated from these logarithmic numbers. Pooled adjusted OR was also calculated. When *I* ^2^ >50%, heterogeneity was significant, and random-effects model was applied to estimate the pooled ORs and 95% CI. We considered *I* ^2^ of <50% as “heterogeneity might not be important,” and a fixed-effects model will be conducted. Cochrane Collaboration's Review Manager Software Package (RevMan 5, Version 5.0, Cochrane Collaboration, Oxford, United Kingdom) was used for the meta-analysis.

## 3. Results

**Figure 1:**
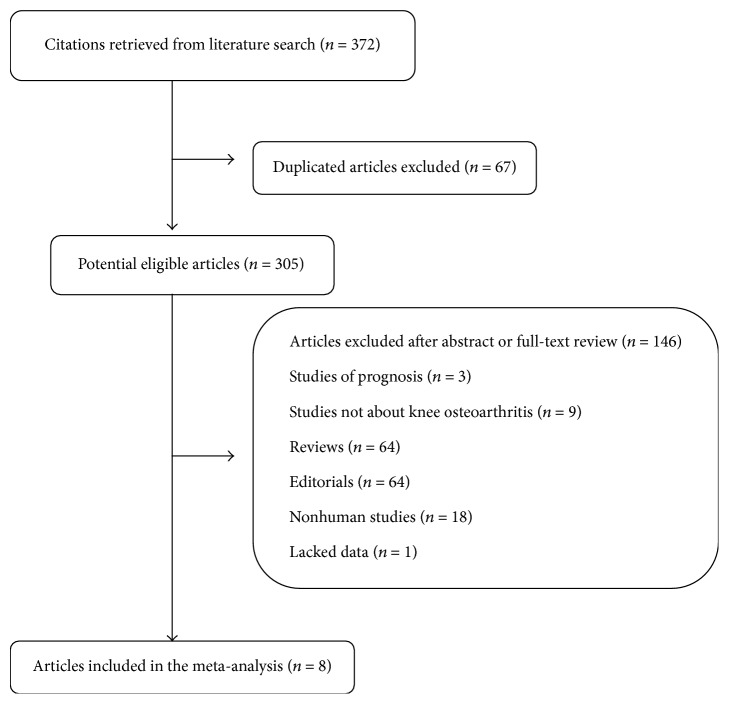
Flow chart of studies selection.

A flow chart of literature search and study selection is shown in Figure 1. After a systematic search in PUBMED, EMBASE, and the Cochrane Library, 371 articles were retrieved. After a systematic review, 8 studies with a total of 3,202 cases and 20,968 controls were finally included. Among these studies, there were two prospective studies [7, 13], three cross-sectional studies [9, 10, 12], and three case-control studies [8, 11]. One study was excluded for lack of data [18]. The characteristics of the included studies are summarized in Table 1.

**Figure 2:**
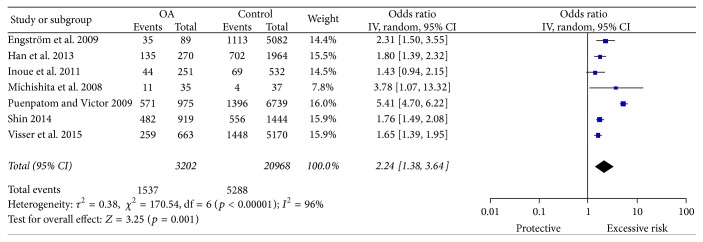
Forest plot of MetS exposure and KOA risk.

**Figure 3:**
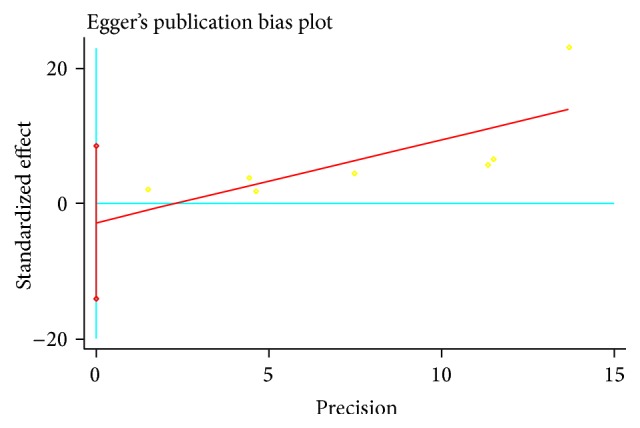
Publication bias evaluated by funnel plots in studies of MetS exposure and KOA risk.

**Figure 4:**
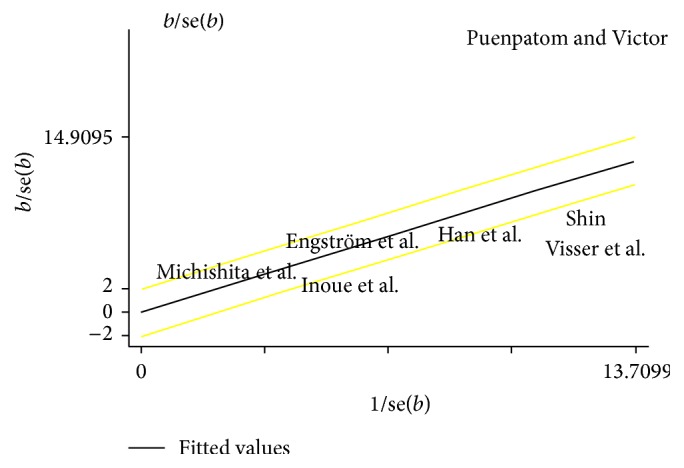
Galbraith plot of ORs of OA.

The pooled ORs result suggested that MetS seemed to significantly increase overall KOA risk (pooled OR = 2.24, 95% CI = 1.38–3.64) (Figure 2). The Egger test did not suggest the presence of publication bias (*p* = 0.555) (Figure 3). However, the individual estimates of the ORs were significantly heterogeneous (*I* ^2^ = 96%, *p* < 0.00001 in random-effects model). The Galbraith plot showed that the study by Puenpatom and Victor largely accounted for heterogeneity (Figure 4). After excluding this study, the interstudy heterogeneity decreased (*I* ^2^ = 0%, *p* = 0.48), with the OR attenuated but remaining statistically significant (pooled OR, 1.74, 95% CI = 1.57–1.92; *p* < 0.00001 in a fixed effect model).

**Figure 5:**
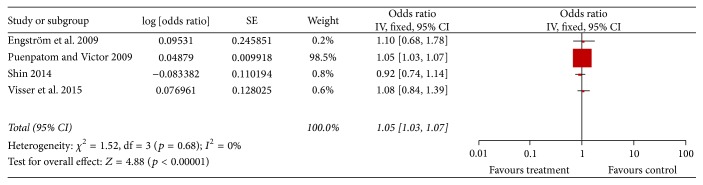
Forest plot of MetS exposure and KOA risk with risk factors adjusted.

**Figure 6:**
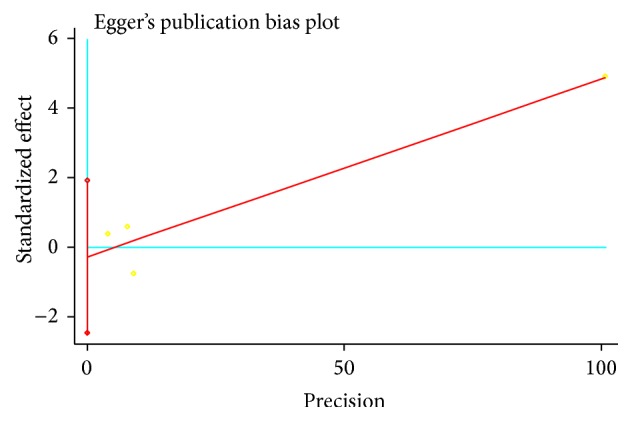
Publication bias evaluated by funnel plots in studies of MetS exposure and KOA risk with risk factors adjusted.

Four studies described the adjusted OR [7, 11–13]. After pooling the adjusted ORs, the results remained significant (pooled adjusted OR = 1.05, 95% CI = 1.03–1.07, and *p* < 0.00001) (Figure 5). No significant interstudy heterogeneity was observed (*I* ^2^ = 0%, *p* for heterogeneity = 0.86) and there was no publication bias (*p* = 0.649) (Figure 6).

## 4. Discussion

KOA is the most prevalent form of arthritis and a major cause of pain and disability that affected 151 million individuals globally in 2000 [1, 2]. It has long been considered a “wear-and-tear” disease leading to loss of cartilage and used to be considered the sole consequence of any process leading to overloading pressure on knee joint. In recent studies, increasing evidence supports that MetS is associated with knee OA [4, 5]. MetS is a clustering of risk factors, including dyslipidemia, high blood pressure, impaired glucose tolerance, and abdominal obesity [15]. It is reported that MetS is prevalent in 59% of patients with OA and in 23% of the population without OA, in a population-based cohort study with a sample of 7,714 people across all ages [15]. Studies also proved that people with MetS develop OA at an earlier age and have more generalized pathology, increased inflammation, and augmented intensive pain in the joints, in comparison with patients with OA in the absence of MetS [4–6, 8, 14–16]. On the contrary, some literature results are controversial as there are other groups that do not support this belief [12]. Thus, it is meaningful to provide a summary and analysis of published studies based on the association between OA and MetS.

This study showed that MetS increases the risk of KOA, even after adjusting many of the risk factors. In our meta-analysis, one article was excluded for not providing the data for analysis. However, in the excluded article, it was indicated that MetS was prevalent in 59% of patients with OA, whereas it was 23% in the population without OA [18]. Thus, excluding this study did not alter the result of the current study. Aging remains the most important risk factor for OA. The overall incidence of knee OA in people at 65+ years of age is reported to be 40%. In the summary of adjusted OR, all studies have age adjusted.

OA and MetS share the same mechanisms of inflammation, oxidative stress, common metabolites, and endothelial dysfunction [5, 19–21]. Obese individuals have a higher risk for OA compared to nonobese people [22]. Metabolites characteristics including energy metabolism and lipid and carbohydrate metabolism in the OA patients' synovial tissue cultures were significantly decreased compared with those with little or no evidence of the disease [23, 24]. Obesity increased mass and joint load and altered proinflammatory factor adipokines secretion, leading to the chronic low grade inflammatory status in joint tissues [25, 26]. Additionally, cholesterol accumulation in the cartilage can impair the efflux function of cartilage, hence inducing OA [27, 28]. Oxidized LDL can activate synovial cells such as macrophages, endothelial cells, and synovial fibroblasts, resulting in release of growth factors, MMP, and proinflammatory cytokines, causing cartilage destruction and bone deformations [29].

As parts of the metabolic syndrome, insulin resistance, hyperglycaemia, and hyperinsulinemia are also strongly related to OA pathogenesis. In clinic, diabetes has been proved to be an independent predictor for osteoarthritis by the population-based cohort study followed over 20 years [30]. There is an increasing recognition of the mechanistic link between OA and DM include advanced glycation endproducts (AGEs), dyslipidemia, adipokines, and cytokines act through oxidative stress and inflammatory mechanisms [24–27]. Dyslipidemia with increased free fatty acid and decreased high density lipoprotein and adiponectin is associated with OA development by decreased vascular reactivity and endothelial dysfunction [31, 32]. Inflammatory transformation and proinflammatory cytokines could result in fibrosis of the synovium through the NF*κ*B signalling pathway which plays a critical role during OA development [19, 33].

All of the above highlight the association between MetS and KOA. MetS not only increases the susceptibility to KOA but also exerts influence on its progression and prognosis. Yoshimura et al. demonstrated not only a dose-response relationship between MetS and KOA but also the relationship between MetS components accumulation and KOA progression and occurrence [15, 34]. Our study results could draw attention to the role of MetS in aetiopathology of KOA and give rise to new potential treatments.

This study has several limitations. First, the number of studies evaluated was very small. Future studies should include a more comprehensive analysis of the topic. Second, although genetic risk factors for OA have been found to contribute to the establishment and progression of this condition [35], in this study, we could not establish the effects of genetic factors and other risk factors potentially influencing MS and KOA.

## 5. Conclusion

For the first time, with meta-analysis, the synthesis of available evidence supports that metabolic syndrome increases the risk for knee osteoarthritis, even after adjustment for many risk factors. As a result, OA is a heterogeneous disease and metabolic factors contribute substantially to its pathogenesis. Furthermore, understanding that MetS contributes to KOA patients will be useful in assessing KOA patients' individual conditions and selection of precision therapeutic strategies.

## Figures and Tables

**Table 1:** Characteristics of studies included in the meta-analysis.

Study	Study design and population	Ethnic	MetS criteria	OA criteria	Number of KOA/control	Age (KOA/control)	Gender	Waist circumference (KOA/control) (cm)	Weight (KOA/control) (kg)	BMI (KOA/control) (kg/m^2^)	Adjusted OR, 95% CI	Adjusted variables
Engström et al. 2009 [7]	Population-based cohort	Sweden	NCEP-ATPIII	A first knee arthroplasty or high tibial osteotomy and diagnosis of OA (715 or M17 according to ICD-9 and ICD-10)	89/5082	(59.7 ± 5.3)/(57.6 ± 6.0)^*∗*^	Male and female	NE	NE	28.9 ± 4.6/25.7 ± 3.9^*∗*^	1.1 (0.7–1.8)	Age, sex (all participants), smoking, CRP, physical activity, BMI
Han et al. 2013 [8]	Case-control	Korean	NCEP-ATPIII	NR	270/1964	(64.5 ± 10.1)/(53.2 ± 11.0)^*∗*^	Male and female	85.0 ± 9.5/82.7 ± 8.7^*∗*^	58.3 ± 9.5/62.0 ± 10.1^*∗*^	24.6 ± 3.3/23.8 ± 3.1^*∗*^	NE	NE
Inoue et al. 2011 [9]	Cross-sectional	Japan	NCEP-ATPIII	K-L grade	251/532	NR	Male and female	Male: 84.5 ± 7.9/84.6 ± 7.4^*∗*^ Female: 84.6 ± 7.4/80.5 ± 8.2^*∗*^	NE	male: 23.6 ± 2.8/23.5 ± 2.7^*∗*^ female: 23.8 ± 3.6/22.3 ± 2.7^*∗*^	NE	NE
Michishita et al. 2008 [10]	Cross-sectional study	Japan	NCEP-ATPIII	K-L grade	35/37	(60.1 ± 6.7)/(58.6 ± 5.3)^#^	female	91.7 ± 8.0/87.2 ± 9.0^*∗*^	68.9 ± 10.0/60.9 ± 8.2^*∗*^	28.2 ± 3.7/26.2 ± 2.8^*∗*^	NE	NE
Puenpatom and Victor 2009 [11]	Case-control	American	NCEP-ATPIII	K-L grade	975/6739	Age 18–65 years: (43.1% versus 92%)^*∗*^ Age >65 years: (56.9%/9.8%)^*∗*^	NE	NE	NE	NE	1.05 (1.03–1.07)	Age, controlled for BMI
Shin 2014 [12]	Cross-sectional study	Korean	NCEP-ATPIII	K-L grade	919/1444	67.2 ± 8.4/61.0 ± 8.1	Male	85.3 ± 8.8/82.2 ± 8.9	61.0 ± 10.2/60.6 ± 9.9	24.7 ± 3.2/23.5 ± 2.9	0.92 (0.74–1.13)	Age, sex, income, smoking, alcohol consumption, physical activity, BMI
Visser et al.2015 [13]	Population-based prospective cohort	Netherlander	NCEP-ATPIII	ACR criteria	663/5170	58/55	Male and female	NE	80.4 ± 12.6/77.8 ± 10.2	26.9 ± 0.08/25.5 ± 2.5	1.08 (0.85–1.39)	Age, sex, smoking, education, ethnicity, height, weight

*∗*: *p* < 0.05 and #: *p* > 0.05; K-L grade: Kellgren-Lawrence grade; NR: not recorded; and NE: not evaluated. Values are presented as mean ± SD.
